# Physicians Experience with and Expectations of the Safety and Tolerability of WHO-Step III Opioids for Chronic (Low) Back Pain: Post Hoc Analysis of Data from a German Cross-Sectional Physician Survey

**DOI:** 10.1155/2015/745048

**Published:** 2015-10-18

**Authors:** Michael A. Ueberall, Alice Eberhardt, Gerhard H. H. Mueller-Schwefe

**Affiliations:** ^1^Institute for Neurological Sciences, Nordostpark 51, 90411 Nuernberg, Germany; ^2^Mundipharma GmbH, Mundipharmastraße 2, 65549 Limburg, Germany; ^3^Interdisciplinary Center for Pain and Palliative Care Medicine, Schillerplatz 8/1, 73033 Goeppingen, Germany

## Abstract

*Objective*. To describe physicians' daily life experience with WHO-step III opioids in the treatment of chronic (low) back pain (CLBP). *Methods*. Post hoc analysis of data from a cross-sectional online survey with 4.283 Germany physicians. *Results*. With a reported median use in 17% of affected patients, WHO-step III opioids play a minor role in treatment of CLBP in daily practice associated with a broad spectrum of positive and negative effects. If prescribed, potent opioids were reported to show clinically relevant effects (such as ≥50% pain relief) in approximately 3 of 4 patients (median 72%). Analgesic effects reported are frequently related with adverse events (AEs). Only 20% of patients were reported to remain free of any AE. Most frequently reported AE was constipation (50%), also graded highest for AE-related daily life restrictions (median 46%). Specific AE countermeasures were reported to be necessary in approximately half of patients (median 45%); nevertheless AE-related premature discontinuation rates reported were high (median 22%). Fentanyl/morphine were the most/least prevalently prescribed potent opioids mentioned (median 20 versus 8%). *Conclusion*. Overall, use of WHO-step III opioids for CLBP is low. AEs, especially constipation, are commonly reported and interfere significantly with analgesic effects in daily practice. Nevertheless, beneficial effects outweigh related AEs in most patients with CLBP.

## 1. Background and Introduction

WHO-step III opioids are increasingly prescribed for the treatment of chronic nonmalignant pain (CNMP) in industrialized countries [1–3]. Based on German health insurance data, overall consumption of WHO-step III opioids by patients suffering from CNMP increased from 24.1 million defined daily doses (DDD) in 2000 to 107.6 million in 2010 [4]. Underlying reason for this 4-fold increment is increases in treatment prevalence (from 0.18 to 0.86% of the German population, mainly due to an increased use in CNMP such as back pain), and treatment duration (e.g., the proportion of patients receiving opioids for longer than 90 days increased from 4.3% in 2000 to 7.5% in 2010) [4].

The increasing popularity of WHO-step III opioids for CNMP is frequently explained by their pharmacological interaction with endogenous pain relieving systems (going hand in hand with efficacy in a broad spectrum of painful conditions), the lack of analgesic ceiling effects (i.e., doses can be escalated until either desired or limiting effects are reached), and the absence of a significant toxicity to internal organs; although side effects from these drugs are common, they are usually transient and reversible upon treatment discontinuation in contrast to those observed with quite a few nonopioid analgesics (e.g., acetaminophen, dipyrone) [5, 6] and most of the nonsteroidal anti-inflammatory drugs (NSAIDs) [7–11].

When used properly, WHO-step III opioids are known to counteract pain that follows surgery, to palliate suffering associated with advanced cancer and to mitigate CNMP. However, while their use for the treatment of postsurgical and cancer-related pain is generally accepted and the available scientific evidence is graded as sufficient to justify their use, the framework for a general recommendation to use them in patients suffering from CNMP is insufficient. Due to the limited evidence from controlled clinical trials, the lack of long-term effectiveness, tolerability, and safety data for treatments lasting longer than three months, and the potential interaction of opioids with endogenous factors leading to individually more or less critical biological, psychological, and social implications, current treatment guidelines uniformly recommend a conservative use of WHO-step III opioids for CNMP [12–16].

Long-term daily use of opioids is associated with significant side effects (e.g., constipation and opioid-induced bowel dysfunction, dizziness, concentration problems, urinary retention, and itching) in the majority of patients, which, even if not associated with a relevant organ toxicity, significantly interfere with patient outcome and pain relieving effects. In addition, a low but nevertheless significant risk exists to develop an opioid use disorder (OUD) characterised by clinical signs/symptoms of either drug abuse, dependence, and/or addiction, even in those patients for whom the treatment indication is based on generally agreed considerations and who take their opioids as directed. Available data suggests that the risk for an OUD varies with patient history, psychosocial factors, and comorbid psychopathology and increases with longer treatment duration, use of immediate release preparations, higher opioid dose, and polymedication [17]. However, even the absence of all these risk-factors does not necessarily eliminate any risk for the development of an OUD.

Moreover, few opioid patients develop an opioid tolerance (probably due to a desensitization and/or downregulation of opioid receptors), in which the ongoing exposure to the drug results in a decline of the primarily achieved effects over time [18]. And beyond that phenomenon (which is usually counteracted either by an increment in opioid dose or by an opioid rotation), opioids might also induce neuroplastic changes in the peripheral and central nervous system (CNS) that lead via a sensitization of pronociceptive pathways to an opioid-induced hyperalgesia [19]. Unfortunately, this complication presents clinically at first sight similarly to the tolerance phenomenon and is consecutively followed by reactive dose escalating countermeasures, which vice versa open the door to a vicious circle of treatment changes that do not only decrease the chance for a beneficial outcome but also increase the risk for an OUD. In combination with the multifactorial reasons underlying CNMP, this complexity of opioid-effects may explain why WHO-step III agents can be remarkably helpful in many patients with CNMP, while in others they either do not work or even increase the problems over time.

In response to the increasing prescription rates of strong acting opioids for patients with CNMP, experts and guidelines critically state that the long-term attested underuse of opioids at the end of the 20th century has now been transformed into a wrongful over- and a critical misuse and that the physicians responsible for that either do not know about the multifractal peculiarities of opioids and related risks in CNMP or even ignore any evidence-based treatment recommendations by overestimating positive and underestimating negative opioid-related effects interfering with patient outcome.

To highlight the silent reproach underlying these statements and to gain further insight into the real-life experience and expectations of physicians treating CNMP patients with WHO-step III opioids, the steering committee of the German Pain League, Germany's largest pain patients self-regulating community, asked the German Pain Association to perform a post hoc analysis of so far unpublished data of a large cross-sectional survey of 4.283 German physicians originally performed in 2012 and to focus now on data addressing global tolerability and safety aspects of treatments with WHO-step III opioids in CNMP.

## 2. Methods

Between August and September 2012, the German Pain Association initiated an online census and invited nearly 12,000 German physicians, known to have a special focus in pain patients and pain therapy, to participate in a cross-sectional national survey entitled CROSSECCO (cross-sectional noninterventional evaluation of physicians' preferences and experiences with strong acting opioid analgesics for the treatment of chronic nonmalignant pain) and to report about their experience with and their expectations of WHO-step III opioids in CNMP. Methodology and results (focusing on general and national aspects) of this survey have been published in 2014 [20]. However, due to the increasing concerns about safety issues of WHO-step III opioids in patients suffering from CNMP [especially chronic (low) back pain, CLBP] and in reaction to the proposal of the German Pain League the German Pain Association commissioned a post hoc analysis of so far unpublished data of this survey focusing now on global tolerability and safety aspects of WHO-step III opioids for the treatment of CLBP that outreach national conditions. The concept for this post hoc analysis has been developed by MAU and GHHMS and has been finally approved by the steering committees of the German Pain Association and the German Pain League.

The original online survey consisted of 157 questions addressing the individual profile of participating physicians (e.g., age, years of experience, specialisation, additional qualifications in pain therapy, and type of practice setting), their patients (e.g., number of patients with pain by condition, duration of pain and stage of chronification, and biological and psychological pain characteristics), and their experience with as well as expectations of various opioids for different pain conditions. Beside general information, a considerable proportion of questions focused on the use of WHO-step III opioids for the treatment of CLBP and addressed patient baseline characteristics (e.g., history, pain duration, intensity, localization, chronification, functional handicaps, restrictions in daily life and quality of life, and comorbidities), pretreatment data, type and duration of opioid treatment (dose, usage characteristics, etc.), opioid-related treatment effects (e.g., pain relief, improvements with respect to quality of life and daily life activities), and safety and tolerability aspects.

Dependent on type of question, participants were asked either to select one of several predefined verbal or numerical options to answer a (multiple-choice type) question or to move a slider on a 100 mm visual analogue scale (VAS, ranging from 0 = none to 100 = all) to quantify the percentage of patients to whom a distinct question or statement belonged.

Statistics were done descriptively only and with respect to the dimensions of the scales used (i.e., for categorical scales absolute and relative (if necessary adjusted) percentages were evaluated and for ordinal scales mean, standard deviation, median, and percentiles). For graphical analyses box-and-whisker diagrams were used (with the bottom and top of the box defined by the first and third quartiles, the band inside by the median, and the whiskers representing the 2.5th and 97.5th percentiles). All analyses were done with the “as reported data set” without any data imputation for missing parameters. Average (±standard deviation) participation rate of physicians per question was 98.9 (±0.8) percent (median 99.1%). Statistical procedures were applied if appropriate to evaluate the significance of differences found not to confirm any predefined hypotheses. Test procedures used were the Chi-Square test for categorical and the Student *t*-test for ordinal scaled variables.

## 3. Results

### 3.1. Relevance of WHO-Step III Opioids for CLBP

**Figure 1:**
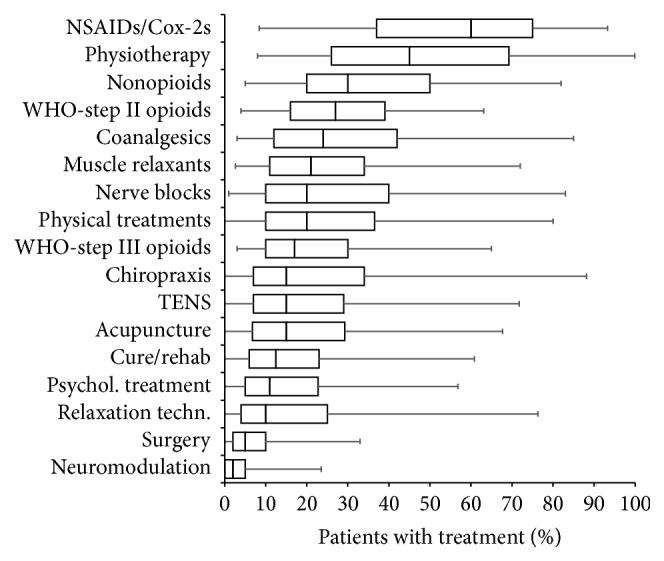
Frequency of use of different pharmacological and nonpharmacological treatment approaches for chronic low back pain sorted by percentage in descending order. Parameters shown are box-and-whisker diagrams (with the bottom and top of the box defined by the first and third quartiles, the band inside by the median, and the whiskers representing the 2.5th and 97.5th percentiles). NSAIDs: nonsteroidal anti-inflammatory agents; Cox-2s: selective Cox-2 inhibitors; WHO: World Health Organization; TENS: transcutaneous electric nerve stimulation.

As reported by the participants of this survey, WHO-step III opioids have a definitive but subordinate meaning among the spectrum of pharmacological and nonpharmacological treatments usually used for patients suffering from CLBP (see Figure 1).

With respect to the spectrum of those 17 treatment approaches for CLBP evaluated in the original survey, reported prescription rates of WHO-step III opioids were low and rank on average (mean ± SD) with 22.7 ± 17.2% (median 17%) on position 9 of the list. Reported prescription rates of NSAIDs/Cox-2s for CLBP were with 55.1 ± 23.8% (median 60%) significantly higher (*p* < 0.001; rank 1), as well as those for nonopioid analgesics (35.6 ± 20.9, median 30%; *p* < 0.001; rank 3), WHO-step II opioids (28.3 ± 16.0, median 27%; *p* = 0.001; rank 4), coanalgesics (29.8 ± 22.3, median 24%; *p* < 0.001; rank 5), muscle relaxants (25.8 ± 18.5, median 21%; *p* < 0.001; rank 6), and selective nerve root blocks/facet joint injections (27.8 ± 23.0, median 20%; *p* < 0.001, rank 7). Nonpharmacological approaches such as physiotherapy and physical treatments were with reported prescription rates of 47.7 ± 25.9% (median 45%; *p* < 0.001; rank 2) and 25.6 ± 20.5% (median 20%; *p* = 0.027; rank 8) which are significantly more commonly used for CLBP patients than WHO-step III opioids, whereas chiropractic procedures (24.5 ± 24.5%, median 15%), transcutaneous electrical nerve stimulation (21.4 ± 19.5%, median 15%), and acupuncture (20.8 ± 19.3%, median 15%) were reported to be comparably often prescribed (*p* = ns).

### 3.2. Opioid Treatment Characteristics

**Figure 2:**
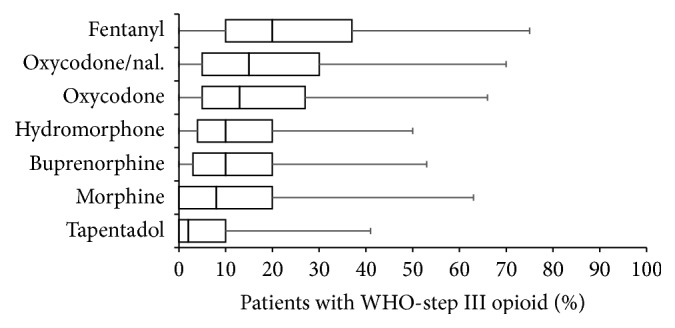
Frequency of use of different WHO-step III opioids for the treatment of chronic low back pain sorted by percentage in descending order. Parameters shown are box-and-whisker diagrams (with the bottom and top of the box defined by the first and third quartiles, the band inside by the median, and the whiskers representing the 2.5th and 97.5th percentiles).

With reported prescription rates of 26.0 ± 20.8% (median 20%) fentanyl was the most frequently used WHO-step III opioid for CLBP (see Figure 2), followed by oxycodone/naloxone (19.9 ± 18.5, median: 15%), oxycodone (17.8 ± 17.1, median 13%), hydromorphone (13.9 ± 13.6, median 10%), buprenorphine (13.8 ± 15.0, median 10%), morphine (13.7 ± 16.8, median 8%), and tapentadol (7.3 ± 12.5, median 2%), a WHO-step III centrally acting analgesic with a dual mode of action (opioid/NA reuptake inhibition).

With 16.5 ± 18.5% (median 11%), physicians reported monotherapy with opioids to be more the exception than the rule. In most patients, survey participants reported to give WHO-step III opioids in combination with other analgesics such as NSAIDs/Cox-2s (23.4 ± 15.8, median 21%), adjuvant agents (21.9 ± 13.7, median 20%), or nonopioid analgesics (18.5 ± 17.6, median 17%). Use of WHO-step III opioids as part of a multimodal treatment concept in combination with several other approaches has been reported for 23.6 ± 17.4% (median 20%) of CLBP patients.

**Figure 3:**
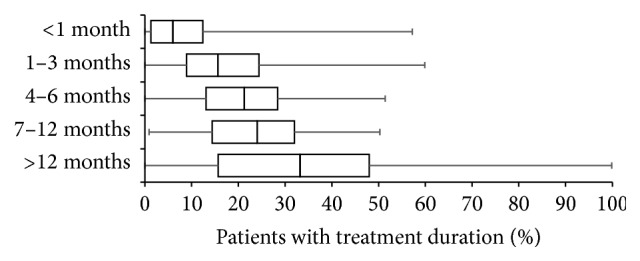
Treatment duration of patients with chronic low back pain with WHO-step III opioids. Parameters shown are box-and-whisker diagrams (with the bottom and top of the box defined by the first and third quartiles, the band inside by the median, and the whiskers representing the 2.5th and 97.5th percentiles).

Treatment duration varied with respect to treatment effects achieved and adverse effects experienced (see below). Average proportion of patients reported to receive WHO-step III opioids for less than 4 weeks was 9.4 ± 10.2% (median 6%), 16.9 ± 10.8% (median 16%) for 1–3 months, 19.4 ± 9.2% (median 21%) for 4–6 months, 21.4 ± 9.8 (median 24%) for 7–12 months, and 32.9 ± 19.4 (median 33%) for treatments longer than 12 months (see Figure 3).

### 3.3. Pain Relief and Related Treatment Effects

Overall, beneficial treatment effects achieved with the introduction of WHO-step III opioids were reported to be satisfying (see Figure 5). A favourable response to the treatment has been reported for 69.6 ± 23.8% of patients (median 75%) and for 67.5 ± 23.7% (median 72%) survey participants reported a pain relief of at least 50% versus pretreatment. Average percentages of patients for whom a clinically relevant improvement with respect to their daily life activities and their overall quality of life has been reported were 64.8 ± 23.6% (median 68%) and 64.6 ± 23.0% (median 70%), respectively. For 54.0 ± 25.3% (median 55%) of CLBP patients, physicians reported that the use of WHO-step III opioids and the pain relief achieved with their introduction paved the way to conduct or participate in alternative treatment approaches; patients were not able to do so before (e.g., physiotherapy, sport, and cure/rehab). For one-third of CLBP patients (38.8 ± 26.8, median 37%), survey physicians reported that the beneficial effects achieved with WHO-step III opioids continued beyond the treatment period and persisted despite treatment discontinuation. Vice versa, for one-third of patients (34.3 ± 25.8, median 29%) significant worsening of pain intensity and related issues has been reported after treatment discontinuation.

### 3.4. Safety and Tolerability Aspects

Adverse effects (AEs) with WHO-step III opioids were reported to be frequent (see Figure 6). On average, transient/short-term AEs (≤2 weeks) were reported for 18.2 ± 11.3 (median 16%) of patients, intermediate AEs (lasting 3–8 weeks) for 44.7 ± 26.1 (median 45%), and persistent/long-term AEs (>8 weeks) for 17.6 ± 9.8% (median 19%). Only for one in five patients (25.3 ± 23.2, median 20%), physicians reported no relevant adverse events in response to the treatment with a WHO-step III opioid.

The most frequently stated AE was constipation with an average reporting rate of 49.1 ± 24.9% (median 50%), followed by somnolence (26.6 ± 19.6, median 21%), dry mouth (22.7 ± 20.2, median 18.5%), reduced performance (17.8 ± 17.2, median 13%), and neurological (15.7 ± 14.7, median 11%) and mental problems (13.3 ± 12.7, median 10%; see Figure 7). Overall, AE-related restrictions were graded highest for constipation (46.1 ± 23.5, median 46%), followed by opioid-induced performance impairments (15.9 ± 17.1, median 10%), urinary retention (11.3 ± 19.8, median 4%), itching (9.2 ± 14.6, median 4%), and endocrine disorders (6.1 ± 11.7, median 1%).

The percentage of patients reported to need specific treatments to countermeasure opioid-related side effects was 58.4 ± 27.4% (median 45%). One-third of those patients (35.6 ± 17.8, median 36%) were reported to put the treatment with WHO-step III opioids generally in question due to the side effects experienced, and one of five patients (20.2 ± 14.8, median 22%) discontinued prematurely. Consecutively, AE-related problems due to WHO-step III opioids are frequently noted: for 6.2 ± 9.8% (median 2%), respectively, 7.3 ± 11.5% (median 3%) of patients, survey participants reported AE-related familial and social problems and emotional and physical distress for further 7.3 ± 12.9% (median 2%) and 8.8 ± 12.5% (median 4%) in addition; 12.3 ± 15.5% (median 7%) of CLBP patients treated with strong opioids were reported to be temporarily, 8.6 ± 13.5% (median 3%), persistently unable to work due to treatment-related AEs, and 3.7 ± 8.2% (median 2%) lost their job due to opioid-related side effects (see Figure 8).

### 3.5. Prevalence of Tolerance, Hyperalgesia, and Opioid Use Disorder

**Figure 4:**
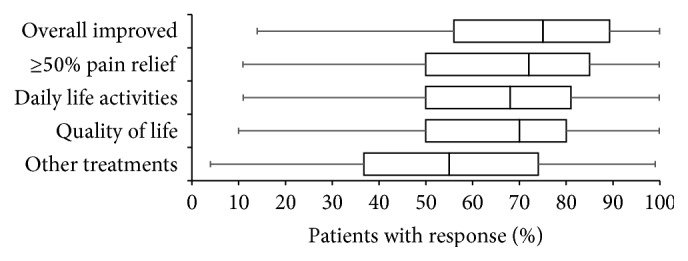
Frequency of different effects reported with WHO-step III opioids in patients with chronic low back pain. Parameters shown are box-and-whisker diagrams (with the bottom and top of the box defined by the first and third quartiles, the band inside by the median, and the whiskers representing the 2.5th and 97.5th percentiles).

**Figure 5:**
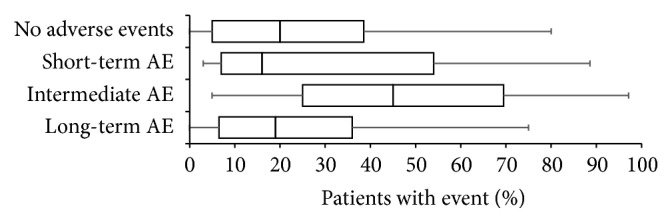
Frequency of different adverse effect experiences reported with WHO-step III opioids used for the treatment of patients with chronic low back pain sorted by percentage in descending order. Parameters shown are box-and-whisker diagrams (with the bottom and top of the box defined by the first and third quartiles, the band inside by the median, and the whiskers representing the 2.5th and 97.5th percentiles). Short-term: duration ≤2 weeks; intermediate term: duration 3–8 weeks; long-term: duration >8 weeks. AE: adverse event.

**Figure 6:**
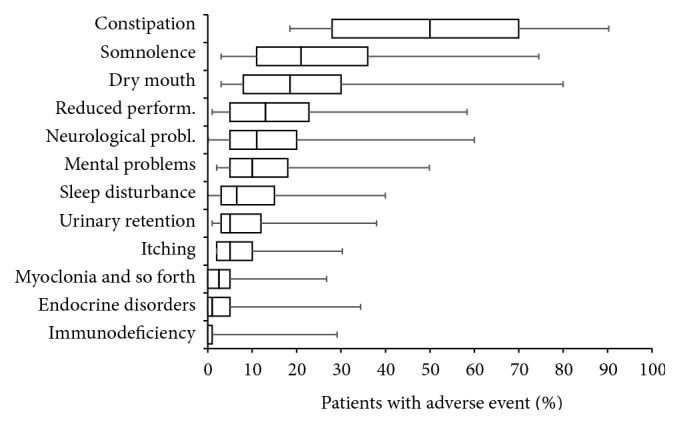
Frequency of different side effects reported with WHO-step III opioids used for the treatment of patients with chronic low back pain sorted by percentage in descending order. Parameters shown are box-and-whisker diagrams (with the bottom and top of the box defined by the first and third quartiles, the band inside by the median, and the whiskers representing the 2.5th and 97.5th percentiles).

**Figure 7:**
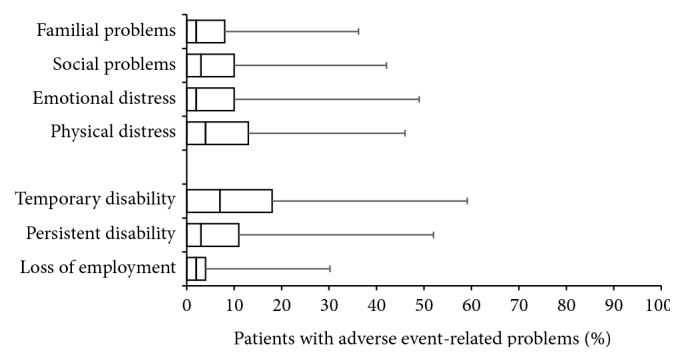
Frequency of different side effect-related complaints reported with WHO-step III opioids used for the treatment of patients with chronic low back pain. Parameters shown are box-and-whisker diagrams (with the bottom and top of the box defined by the first and third quartiles, the band inside by the median, and the whiskers representing the 2.5th and 97.5th percentiles).

**Figure 8:**
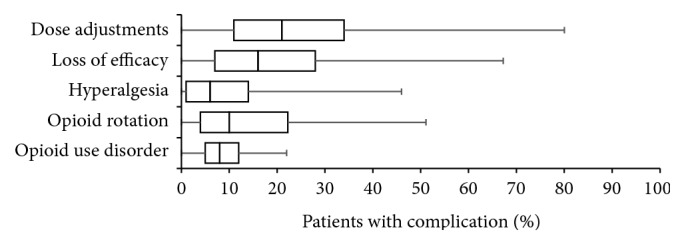
Frequency of different application problems reported with WHO-step III opioids for the treatment of patients with chronic low back pain. Parameters shown are box-and-whisker diagrams (with the bottom and top of the box defined by the first and third quartiles, the band inside by the median, and the whiskers representing the 2.5th and 97.5th percentiles).

On average, survey participants reported for one-fourth of patients (26.2 ± 19.9, median 21%) the need for recurrent dose adjustments during opioid maintenance treatment to keep analgesic effects stable (see Figure 4). Nevertheless, for one in five patients (20.2 ± 17.9, median 16%) a loss of efficacy over time and for one of six patients (15.1 ± 14.6, median 10%) an opioid rotation to keep treatment effects stable have been reported. Reported percentage of patients who develop an opioid-induced hyperalgesia was 10.4 ± 13.1% (median 6%).

For 9.1 ± 6.0% (median 8%) of CLBP patients, physicians reported the development of signs and clinical correlates suggestive for an opioid use disorder.

## 4. Discussion

Use of WHO-step III opioid analgesics for the management of CNMP and especially CLBP has increased dramatically over the past decade with corresponding increases in negative sequelae and rising concerns about these developments worldwide. Beside pure reaction to the growing population of patients suffering from both conditions, experts frequently criticise an uncritical overprescription of strong opioids by physicians due to an overestimation of beneficial effects, whitewashing of tolerability problems, and denial of critical safety issues.

However, the current analysis of data from a cross-sectional German physician survey shows a slightly different picture:

 With a median prescription rate of 17%, treatment with WHO-step III opioids is more the exception than the rule for patients suffering from CLBP.
 In four of five patients, opioids are combined with other pharmacological treatments and nonpharmacological approaches and in 20% as part of a multimodal pain management regimen.
 Treatment duration is reported to be highly individualized dependent on the extent of beneficial effects and tolerability issues. Nevertheless about one-third of patients received WHO-step III opioids for more than 12 months.
 Overall, treatment effects are substantial, but far below overwhelming. For seven of ten patients, survey participants noted a clinically relevant improvement with respect to their overall situation and for two-thirds of patients they reported significant treatment-related changes, for either pain relief, improvement in daily life activities, or quality of life.
 Opioid-related side effects are frequent (only 1 in 5 patients is reported to develop none), are associated with relevant emotional and physical distress (1 in 6), interfere significantly with familial and social life (1 in 10), and lead to transient or persistent disability (1 in 10 and 1 in 16) or even complete loss of employment (1 in 30).
 Proportion of patients with tolerance development and the need for dose adjustments during maintenance treatment (reported for 1 in 4 patients) is rather high, as well as the percentages of patients who present with an untreatable loss of efficacy (1 in 5), those who need an opioid rotation (1 in 6), and those who develop an opioid-induced hypersensitivity (1 in 10).
 With 9 in 100, the proportion of patients with clinical signs suggestive for an opioid use disorder is low, but clinically relevant.

Due to these data, the prescription of WHO-step III opioids to patients with CLBP is obviously not primarily driven by an overestimation of effects and/or a related underestimation of side effects. Reported tolerability and safety data is close to those given in the current literature and particularly the proportion of patients seen at risk for the development of tolerance (treatable or not), opioid-induced hypersensitivity, and OUD by the survey participants have to be taken as a correlate that the prescription of opioids occurs usually being well reflected and with full awareness for critical opioid-related risk factors by the physicians involved.

Differences between reported effect sizes and effect rates in this survey versus those published for RCTs are predominantly due to the methodological differences of artificial settings under study versus real-life conditions. Compared with data from noninterventional trials with WHO-step III opioids in CLBP, reported pain relief and related effects on daily function as well as quality of life are comparable [21, 22]. Nevertheless, reported efficacy expectations are high and reflect probably not only objectively verifiable effects, but also related expectations.

As shown by the low percentage of WHO-step III prescriptions, strong acting opioids are more the last than the first choice for patients with CLBP. It can be therefore assumed that physicians prescribe these agents to those CLBP patients only, who showed either none or only insufficient analgesic effects in response to alternative approaches or to those with intolerable side effects of previous pharmacological measures. Under these circumstances, the absolute risk-balance between positive and negative effects of a distinct treatment transforms significantly and becomes relative. If nothing works sufficiently well or well enough to be maintained, or in case prior treatments have to be stopped due to intolerable side effects, physicians are forced to choose alternative measures. In such a situation, strong acting opioids seem, for a number of reasons, as potent alternatives and are worth being considered even for patients with so-called nonmalignant (better noncancer) chronic pain, such as CLBP.

With an average/median of 8.1/7 percent, the proportion of patients with WHO-step III opioids reported in this survey to develop an opioid use disorder is in the middle range of previous reports on this issue. In 1986, Portenoy and Foley reported an estimated addiction risk during chronic opioid treatment in patients with nonmalignant pain of around 5% on average [23], whereas Fishbain et al. found in a systematic review of 24 articles a prevalence percentage for drug abuse, drug dependence, and drug addiction in the range of 3.2–18.9% [24]. In addition to a number of methodological differences between both studies and especially to this survey, the currently discussed estimates of addiction risks should be taken with caution, as the reported differences predominantly reflect the lack of a uniquely agreed and valid definition of iatrogenic opioid addiction (i.e., addiction arising during opioid treatment of pain). So far, most of these data reflect a significant spectrum of different definitions and classifications of opioid addiction or opioid use disorder and in most cases the comparability of different risk estimates reported in different studies is rather low, as “iatrogenic opioid addiction” is, what the reporting person says it is, and not what a uniquely agreed consensus statement defines. Nevertheless, the ~9% prevalence of OUD reported in this survey should be taken as a serious indicator for a significant and probably continuously increasing problem, especially in front of the data of an international working group that reported a nearly threefold increase in OUD-related mortality between 1990 and 2013 [25].

Careful patient selection, variable dose titration, individualized treatment durations, ongoing effect evaluations, knowledge about the broad spectrum of opioid-related side effects, and an increased awareness of physiological as well as psychological complications (such as tolerance development, opioid-induced hypersensitivity, and addiction/opioid use disorder) are essential parameters characterizing a patient-centred use of WHO-step III opioids. Data of this cross-sectional physician survey indicate that these key features build (more or less) the fundamentals for a treatment trial with strong opioids. Physicians obviously know about the overall limited efficacy of these drugs and they realize the different tolerability and critical safety risks associated with them. But what kind of alternatives do they have? For more than half of the patients treated with opioids, survey participants reported that “they weren't treatable otherwise.” Dramatically increasing numbers of surgical spine interventions and exploding proportions of patients with consecutive failed back surgery syndromes can be taken as additional indicators for the challenging situation; physicians have to manage in case they are confronted with a patient suffering from CLBP in the 21st century.

Increasing numbers of studies, reports, consensus statements, and guidelines recommend using WHO-step III opioids with knowledge, experience, and caution as well as in a patient-physician relationship built on confidence, partnership, candidness, and time. These factors are critical for the ideal treatment of patients suffering from CLBP. They are essential to tailor different multimodal treatment concepts to the individual needs of affected patients. And they are important to optimize safety, tolerability, and efficacy of potent drugs such as the WHO-step III opioids. If these conditions are clear, guaranteed and agreed between all treatment partners, WHO-step III opioids are a welcome addition to conventional treatment approaches and an important and safe alternative for many patients suffering from CLBP. If not, WHO-step III opioids carry a significant risk to corrupt all other well-intentioned approaches, to worsen the underlying situation, and to harm the patient.

As other studies have shown before and databases currently report, the WHO-step III prescription behaviour of German physicians differs significantly from those of other countries, especially the US. Due to the data of the Pain & Policy Studies Group of the University of Wisconsin and the WHO Collaborating Center, Germany has worldwide the highest per capita fentanyl consumption rate (5.7 mg; rank #1) versus only 1.7 mg in the US (rank #10) [26]. This is also confirmed by our own observation that the transdermal fentanyl patch is the most prevalently prescribed WHO-step III opioid for CNMP in Germany. Reasons for that have been widely discussed and so far no easy answer can be given to explain these discrepancies between the US and Germany. From our point of view, the major reason for the preferential use of fentanyl in Germany is not the opioid compound per se, but the drug-delivery system via a transdermal patch. This combination of an apparently innocent and easy-to-use system (the patch) with a potent opioid (fentanyl) has obviously a significant charm for physicians, nursing staff, and patients in Germany.

## 5. Strength, Weaknesses, and Limitations

Obviously, the cross-sectional survey generating the data for this post hoc analysis as well as this analysis suffers from several methodological limitations that should be taken into account when interpreting these results. Typical for such a survey, data were collected retrospectively, reflect personal experience (e.g., internal evidence), and ground predominantly on spontaneous approximations of data about various clinical and pharmacological parameters. Subsequent imprecisions in estimating the percentages for distinct survey parameters are reflected by the high standard deviations observed for the means and the broad spectrum of VAS scores given. Therefore, average values reported in this study must be interpreted with care and only in conjunction with the dispersion data presented in the corresponding box-plots. A prospective observational study design would allow for more accuracy and use of direct measures, of both efficacy (e.g., pain reduction) and tolerability (e.g., assessment of side effects), but would restrict the number of participants (and by that the representativeness of the data) and vice versa increase costs significantly. To get meaningful information from a larger cohort of physicians and to encourage them to participate, the chosen format of a cross-sectional online survey fulfills all prerequisites for an unbiased acquisition of routine data reflecting daily practice.

The large number of participants is one of the major strengths of the survey underlying this post hoc analysis. With 4.283 participants, this physician survey is one of largest ones performed worldwide and despite the considerable data variation, this sample size guarantees a solid basis for any conclusions drawn.

A critical weakness of this survey is the lack of any differential data assessment with respect to the spectrum of currently available and prescribed WHO-step III opioids for CLBP. Therefore all effects, side effects, and safety issues reported and discussed are those for the whole group of strong acting opioids used. Data on preferred prescription rates point out that there might be some safety and efficacy differences between distinct WHO-step III opioids, but with conventional analysis techniques these differences are not evaluable. Nevertheless, the fact that morphine (the so-called gold standard of opioid treatment) is, due to the data of this survey, the least frequently prescribed WHO-step III opioid for patients with CLBP shows that pharmacological advantages and disadvantages of distinct strong acting opioids directly translate into daily use.

## 6. Conclusion

Based on the data from a large cross-sectional survey among 4.283 German physicians, WHO-step III opioids are used as third-line treatment in patients with CLBP, especially in cases where alternative pharmacological and nonpharmacological approaches failed or are associated with significant risks. Treatment characteristics, as well as information given on effect size, side effects, and safety aspects, give insight into a complex multifractal health problem in which opioids represent only one option among several others. Opioid treatment is based on rational thoughts and realistic assumptions with respect to prevalence and size of positive as well as negative effects. Despite a significant percentage of patients with opioid-related side effects, tolerance development, and even signs of OUD, the overall risk-benefit analysis remains positive and in the majority of patients treated with WHO-step III opioids, treatment-related benefits outweigh associated adverse effects.
